# Periodontal Ecosystem and Clinical Implications of the Oral Microbiome: A Narrative Review

**DOI:** 10.1155/ijod/1479982

**Published:** 2026-06-05

**Authors:** Ana Júlia Martins Targino, Josy Lorena Peres da Silva Vilarinho, Valéria Martins de Araújo Carneiro, Bruna Frizon Greggianin, Maria do Carmo Machado Guimarães

**Affiliations:** ^1^ Department of Dentistry, School of Health Sciences, University of Brasilia, Brasília, Brazil, unb.br

**Keywords:** dysbiosis, host microbial interactions, microbiome, microbiota, periodontitis

## Abstract

**Background:**

Periodontal disease, although widely documented, remains one of the leading causes of tooth loss worldwide. This situation can be attributed to patients’ difficulty in following treatment protocols and to an incomplete understanding of the disease’s mechanisms, which could be explored to develop new therapies.

**Objective:**

The present study aimed to summarize the main advances in understanding the periodontal microbiome, especially concerning the development of genetic sequencing technologies and metabolite analysis, as well as new treatment methods that have emerged from these innovations.

**Methods:**

A narrative literature review was conducted through searches in the PubMed, Scopus, Web of Science, and SciELO databases, prioritizing publications from the past 15 years while also including classic and foundational studies relevant to the theoretical framework.

**Conclusions:**

Periodontal microbiome results from the synergistic interaction among different microorganisms, rather than just the sum of their individual metabolites. This synergy can create structural microarrangements that resist biofilm disruption, such as fungal barriers surrounding Gram‐negative bacteria. Innovative treatments—such as host response modulation with resolvins and oral microbiome modulation, particularly using prebiotics derived from plant extracts (nitrate metabolism)—offer promising prospects. However, limitations remain regarding their clinical use and the challenge of managing refractory cases, highlighting the need for further research to support these findings.

## 1. Introduction

Periodontal disease is a chronic inflammatory condition of multifactorial origin, linked to the presence of dysbiotic biofilms. It is marked by the progressive breakdown of the periodontal tissues that support the teeth [1]. Over the years, advances in understanding the etiopathogenesis of periodontal diseases have influenced the approaches used for their management.

Initially, the nonspecific plaque hypothesis [2, 3] attributed periodontal destruction to the indiscriminate accumulation of microorganisms, leading to treatments focused on mechanical plaque removal and oral hygiene. Subsequently, the specific plaque hypothesis [4] identified key pathogenic species, promoting the use of targeted antimicrobial therapies alongside mechanical plaque control. In the 1990s, Socransky’s microbial complexes [5] highlighted the importance of ecological interactions and microbial succession in pathogenesis, broadening the clinical focus to the reduction of pathogenic microbial groups rather than isolated species. The development of these theories has proven to be complementary over the years, providing a solid foundation for the emergence of more precise and detailed concepts in the future, supported by culture‐independent technologies through genetic sequencing.

With the advent of high‐throughput genetic sequencing, more integrative models emerged, such as the polymicrobial synergy and dysbiosis (PSD) model [6], which interprets periodontitis as a polymicrobial and synergistic disease caused by dysbiosis. In this context, the focus shifts to understanding the sum of interactions among species and their ecosystem as the target of treatment strategies, highlighting the relevance of interventions such as probiotics, prebiotics, and modulation of the host immune response.

The “Inflammation‐Mediated‐Polymicrobial‐Emergence and Dysbiotic‐Exacerbation” (IMPEDE) model [7] identifies inflammation as the driving force behind periodontal dysbiosis, reinforcing the ecological concept proposed by Marsh in 1994, which posits that environmental and host factors favor the enrichment of pathogenic bacteria. This perspective underscores the importance of treatment strategies that not only eliminate microorganisms but also restore the microbial balance and modulate the host immune response [8].

More recently, advances in genetic sequencing technologies and metabolite analysis have allowed for a more comprehensive and detailed understanding of the periodontal microbiome. This narrative review aims to synthesize the main recent developments in understanding the periodontal microbiome, identify promising new therapeutic approaches documented in the literature, and highlight the remaining gaps in current knowledge on the topic.

## 2. Methodology

This study was carried out through a narrative literature review to identify and analyze the main advances related to the periodontal microbiome and the therapeutic innovations stemming from new genetic sequencing technologies and metabolite analysis. A search for scientific articles was performed in the PubMed, Scopus, Web of Science, Lilacs, SciELO, and Google Scholar databases, focusing on publications from the last 15 years, although classical studies were included when considered relevant for the theoretical context. The included articles consisted of clinical trials, randomized controlled trials, systematic reviews, narrative reviews, and scoping reviews. The search strategy used was (“periodontitis”[MeSH Terms] OR “periodontitis” OR “periodontal disease” OR “periodontal diseases”) AND (“understanding” OR “comprehension”) AND (“oral microbiome” OR “microbiome, oral” OR “oral microbiota” OR “microbiota, oral”) AND (“dysbiosis” OR “dysbiosis, microbial” OR “microbial dysbiosis”) AND (“host response” OR “host responses” OR “host‐response”) AND (“pathogenesis” OR “pathogenesis, disease” OR “disease pathogenesis”). In addition, some articles were included based on the authors’ free discretion, following the criteria of relevance within the exposed theme and the year of publication. The included articles were systematic and narrative literature reviews, clinical studies, and observational studies. Texts that were inaccessible, incomplete, or did not fit the scope of the review were excluded.

### 2.1. Classical Microbial Theories

Understanding the causes of a disease is vital for determining the proper treatment approach. In this context, the earliest microbiological theories on periodontal disease focused on identifying specific etiological agents—mainly bacteria—that are linked to the appearance of clinical signs of this condition. An example is the specific plaque hypothesis [4], which suggests that the development of the disease is linked to the growth of certain bacteria within the oral microbiota.

In the 1980s, the idea of what are now called pathobionts—native microorganisms that can cause disease in the host when conditions are right—started to develop [9].

By the 1990s, the microbial complexes model proposed by Socransky gained recognition. According to this model, the subgingival microbiome can be organized into complexes, each containing microorganisms with specific roles, such as colonization, protection, or pathogenicity [5]. This paradigm marked the start of a more detailed understanding of the microbial environment, shifting the perspective from bacteria solely causing harm to recognizing that they can also participate in bacterial succession and serve protective functions.

In 2003, the idea of periodontal disease as a consequence of a breakdown of homeostasis and subsequent microbiota alterations gained attention in the academic community, as had been anticipated by Marsh’s 1994 ecological plaque hypothesis [8]. According to this theory, environmental factors would play a regulatory role, altering the dynamics and composition of the oral microbiome. At that time, the role of the host in disease progression also became evident, since environmental factors are often influenced by the habits or conditions of individuals with periodontitis, such as underlying systemic diseases (e.g., diabetes) or smoking [10].

The traditional view of periodontal microbiology is much broader and more complex than what can be fully covered here. Still, it is important to mention some microbial models as their findings have been supported by genomic studies and are key to developing new microbial theories.

### 2.2. Community‐Level Microbial Interactions in Periodontal Pathophysiology

The development of genetic sequencing technologies using 16S ribosomal RNA and its mapping within the Human Microbiome Project has expanded the understanding of microorganisms involved in the etiopathogenesis of periodontal disease. Mapping the metabolites released and utilized by these microorganisms has, in turn, clarified metabolic pathways important for the progression of periodontal diseases [11].

Over the past decades, studies have focused on characterizing the periodontal microbiome under different health and disease conditions, identifying prokaryotic microorganisms as the most abundant in the oral cavity, with ~700 bacterial species making up the subgingival plaque [12]. In addition to bacteria, fungi, viruses, and archaea are also part of the oral microbiota. Microorganisms that make up the oral microbiome, whether in healthy or pathological states, maintain a certain stability during the transition between these states within an individual. What changes, however, is the community’s total biomass, which tends to increase along with the relative frequency of specific microorganisms. In this context, a healthy oral microbiome shows greater diversity and lower abundance compared with the periodontal microbiome in diseased conditions. In the latter situation, there is not necessarily the emergence of new bacterial species but rather the proliferation of bacteria already present, favoring the formation of more virulent bacterial conglomerates [13].

Oral bacteria, in turn, have shown more versatility than previously thought. Besides the well‐known groups of commensal bacteria and pathobionts, the group called core bacteria has also emerged. The most interesting part is that these bacteria make up a significant portion of the microbiome, maintaining stability in their relative abundance—meaning their proportion compared to the entire bacterial community remains consistent [14, 15]. What sets their role in health and disease apart is their metabolic pathways, which adopt a more proinflammatory role during the disease.

In this context, although the presence of specific bacteria is not directly linked to disease development—as evidenced by individuals carrying bacteria strongly associated with periodontitis without developing the condition—in susceptible individuals, keystone pathogens can alter the oral microenvironment, undermine the host immune system, and promote changes such as increased flagellar motility, bacterial coaggregation, and other effects [16]. These changes support the idea that the various microorganisms in the oral cavity do not associate randomly but instead form conglomerates with protective and synergistic characteristics, which can increase the stability and virulence of these microbial communities, as will be discussed in detail in the section “Microbial Biogeography.”

Another important piece of evidence emphasizing the significance of bacterial conglomerates—in contrast with isolated microorganisms—in the development of periodontal diseases is the quick return of microbial balance after systemic antimicrobial treatment. While improvements in probing depth and gingival bleeding indices can be seen, the periodontal microbiome can reestablish itself within about 3 months. In other words, systemic antibiotics have a notably short‐lived impact on the selection of oral microorganisms. This can be explained by the presence of synergistic metabolic pathways and long‐term feedback loops created by oral microorganisms, which help stably alter the microenvironment [16, 17].

While metabolomic studies highlight the importance of understanding the overall periodontal microbiome and allow validation of pathogenic mechanisms, previous research supports a broader investigation of microorganisms capable of shifting this microbiome toward disease. Therefore, we aim to summarize the main findings from both sources of knowledge, providing a clear understanding of the etiopathogenesis of periodontal diseases.

### 2.3. Periodontal Biogeography

Recent advances in sequencing technologies have substantially expanded the understanding of the periodontal microbiome, enabling a shift from reductionist models toward ecological and systems‐based frameworks. Within this context, the PSD model proposed by Hajishengallis and Lamont [6] has become a dominant paradigm for explaining periodontal disease. However, despite its conceptual strength, the PSD model remains largely descriptive, with important gaps regarding the temporal dynamics and causal hierarchy underlying microbial shifts. In parallel, the concept of microbial biogeography has refined the interpretation of subgingival microbial organization by emphasizing spatial heterogeneity as a determinant of ecological interactions [18], although its integration into clinical frameworks remains limited.

Rather than exhaustively reviewing established knowledge, this section focuses on aspects that remain insufficiently resolved, including spatial organization, ecological succession, and the functional plasticity of microbial communities in response to environmental pressures. In particular, the extent to which microbial shifts precede, follow, or coevolve with host inflammatory responses remains unclear, thereby highlighting a persistent gap in establishing causality in periodontal disease.

Periodontal disease is currently understood as the outcome of a synergistic and dysbiotic polymicrobial community, in which pathogenicity emerges from collective behavior rather than individual species [19]. While this framework has replaced earlier pathogen‐centric models, it simultaneously introduces challenges, particularly in defining specific microbial signatures predictive of disease progression. Early colonizers, predominantly Gram‐positive facultative anaerobes such as *Streptococcus* spp. (e.g., *S. sanguinis* and *S. gordonii*) and *Actinomyces* spp., are consistently associated with health and biofilm stability [16]. However, their role should not be interpreted as purely protective since these organisms actively shape the microenvironment and enable subsequent colonization.

Furthermore, intermediate or core species complicate this framework by exhibiting marked functional adaptability. Through metabolic interactions with both early colonizers and pathobionts, these microorganisms can support either homeostasis or dysbiosis depending on the environmental conditions. Although this ecological plasticity is widely acknowledged [20–22], it remains insufficiently characterized at a mechanistic level, particularly regarding the thresholds that trigger the transition toward disease.

In more advanced stages, pathobionts become increasingly prominent within the microbial community. Nevertheless, their role is better understood in terms of ecological influence than absolute abundance. Keystone pathogens, even at low levels, can disproportionately modulate the microbial ecosystem; notably, *Porphyromonas gingivalis* has been shown to orchestrate inflammatory responses and reshape the community structure [19]. However, emerging evidence suggests that this keystone paradigm may not be restricted to a single species. For instance, *Filifactor alocis*, a Gram‐positive anaerobe increasingly associated with periodontal severity, has recently been proposed as a potential marker of disease progression. Clinical data indicate that its abundance is significantly higher in advanced stages of periodontitis and correlates positively with probing pocket depth and clinical attachment loss, with increases in these parameters explaining up to 23.9% and 14.9% of its concentration, respectively [23]. Moreover, its bacterial load has demonstrated discriminatory capacity between stage III and IV periodontitis, reinforcing its potential role in disease stratification. Importantly, these findings suggest that *F. alocis* may contribute to dysbiosis and inflammatory amplification in a manner functionally analogous to established keystone pathogens. Consequently, periodontal dysbiosis may be driven by a broader network of keystone‐like organisms rather than a single dominant species, thereby expanding the current interpretation of the PSD model proposed by Hajishengallis and Lamont [6] in 2012. Nevertheless, whether *F. alocis* acts as a true initiator of dysbiosis or predominantly as an amplifier within an already inflamed environment remains to be elucidated.

More recently, the IMPEDE framework has extended polymicrobial concepts by positioning inflammation as a primary driver of dysbiosis [7]. While this model offers a compelling inversion of causality, it also raises unresolved questions regarding the initial triggers of inflammation and the bidirectional feedback loops between the host and microbiome [7, 24]. Thus, these limitations underscore the need for integrative approaches capable of disentangling microbial and host contributions.

Although consistent microbial patterns have been associated with periodontal health and disease [13], their predictive value remains limited. For instance, transitional states such as gingivitis are marked by an increased abundance of Gram‐negative taxa, including *Treponema* and *Prevotella* [25]. However, these shifts alone are not sufficient to reliably predict disease progression, thereby reinforcing the multifactorial nature of periodontitis.

From a spatial perspective, the subgingival biofilm displays a highly organized architecture shaped by ecological gradients. In this context, early colonizers such as *Streptococcus* spp. and *Actinomyces* spp. contribute to oxygen depletion, thereby facilitating the establishment of anaerobic niches. Subsequently, bridging organisms, particularly *Fusobacterium nucleatum*, mediate coaggregation and structural integration, while strict anaerobes including *P. gingivalis*, *Tannerella forsythia*, and *Treponema denticola* are preferentially localized in deeper regions. Although this spatial stratification supports metabolic cooperation and functional specialization [21, 26, 27], it also complicates therapeutic disruption, since distinct niches may respond heterogeneously to intervention.

These spatial dynamics are closely linked to microbial succession and metabolic reprogramming. Specifically, the shift from carbohydrate metabolism to proteolytic and amino acid‐based pathways, along with the increased production of short‐chain fatty acids such as butyrate and propionate, reflects adaptation to an inflammatory environment. At the same time, the enrichment of virulence‐associated functions, including lipopolysaccharide (LPS) biosynthesis and immune modulation, suggests that dysbiosis is not merely compositional but also functional. Nevertheless, whether these metabolic changes act as drivers or consequences of inflammation remains unresolved [6, 7, 13, 28].

Importantly, the implications of these processes extend beyond the periodontal niche. The persistence of a dysbiotic and spatially organized biofilm contributes to a sustained low‐grade inflammatory burden, which is closely associated with systemic conditions such as type 2 diabetes mellitus (T2DM) [6, 13, 29]. In turn, this bidirectional relationship further complicates disease management, as systemic factors may continuously reinforce local dysbiosis.

In this context, periodontal therapy cannot be adequately conceptualized as a one‐time intervention. Instead, the structural resilience of the biofilm, combined with its ecological adaptability and integration with host inflammatory pathways, supports the need for continuous management. Supportive periodontal therapy is therefore essential not only to mechanically disrupt biofilm organization but also to modulate the cyclical processes of microbial succession and inflammation [17, 25, 30]. Without sustained intervention, the reestablishment of dysbiotic communities is likely, particularly in patients with underlying systemic conditions, thereby reinforcing the chronic and relapsing nature of periodontitis.

### 2.4. Systemic Diseases and Periodontitis

The contemporary understanding of periodontitis as a chronic dysbiotic inflammatory disease situates it within the broader framework of systemic noncommunicable conditions [1, 31]. No longer confined to the oral cavity, periodontal inflammation is increasingly recognized as a contributor to systemic low‐grade inflammation, immune dysregulation, and metabolic imbalance. This integrative perspective reframes periodontitis as a biologically active condition that can influence distant organ systems [32].

The bidirectional relationship between T2DM and periodontitis has been widely recognized for decades, particularly following the seminal work of Harald Löe, in which periodontal disease was described as the sixth complication of diabetes, alongside classical microvascular alterations [33]. Over the years, this concept has been substantially expanded, evolving from an epidemiological association to a biologically plausible and mechanistically supported model. In this context, chronic hyperglycemia contributes to periodontal tissue breakdown through pathways such as the formation of advanced glycation end products (AGEs) and the upregulation of proinflammatory mediators, including IL‐1β, IL‐6, and TNF‐α [34]. Conversely, periodontal inflammation may impair glycemic control by promoting systemic inflammation and insulin resistance, thereby reinforcing a bidirectional and self‐sustaining cycle. Supporting this perspective, robust evidence from randomized clinical trials and meta‐analyses demonstrates that nonsurgical periodontal therapy—particularly scaling and root planing—can significantly reduce HbA1c levels and systemic inflammatory markers, such as C‐reactive protein, suggesting that periodontal control may positively influence metabolic and cardiovascular risk profiles [35–37].

In addition to its effects on inflammatory pathways, diabetes exerts a profound impact on bone metabolism, further aggravating periodontal breakdown. Chronic hyperglycemia disrupts the balance between bone formation and resorption by impairing osteoblast differentiation and activity while promoting osteoclastogenesis, largely mediated by increased expression of proinflammatory cytokines and receptor activator of nuclear factor kappa‐B (NF‐κB) ligand (RANKL) [38]. This imbalance is further exacerbated by alterations in the osteoprotegerin (OPG)/RANKL axis, favoring a proresorptive environment. AGEs, acting through their receptor (RAGE), contribute to uncoupled bone remodeling by enhancing inflammation and inhibiting bone formation [39, 40]. Moreover, diabetes negatively affects the regenerative capacity of periodontal tissues by impairing the mesenchymal stem cell function, shifting periodontal ligament stem cell differentiation from osteogenic toward adipogenic lineages [41]. Collectively, these alterations compromise bone homeostasis and repair, reinforcing the susceptibility to periodontal tissue destruction in individuals with diabetes [41].

In addition to host‐mediated mechanisms, growing evidence indicates that diabetes is associated with distinct alterations in the subgingival microbiome, further contributing to periodontal disease susceptibility and progression. Dysbiotic microbial communities remain central to periodontitis pathogenesis; however, in individuals with T2DM, this dysbiosis appears to be modulated by the hyperglycemic environment and altered immune response [42]. Recent findings demonstrate that the shift in the subgingival microbiome from health to disease is less pronounced in individuals with T2DM compared with nondiabetic subjects, despite similar clinical periodontal conditions, suggesting a pre‐existing microbial and immunological susceptibility in diabetic individuals. In this context, taxa such as *Eubacterium nodatum*, *Filifactor*, *Fretibacterium*, and *Desulfovibrio* have been implicated in the pathogenesis of periodontitis under diabetic conditions, alongside significant alterations in microbial metabolic pathways, including butyrate and amino acid metabolism [43]. These changes occur in parallel with immune dysregulation, characterized by an imbalance in T‐cell subsets—particularly a reduced Treg/Th17 ratio—and increased expression of immune checkpoint markers, reinforcing a permissive environment for chronic inflammation. Notably, the relatively small differences observed between the microbiome of diabetic individuals with and without periodontitis further support the concept that diabetes itself may predispose to periodontal breakdown, as previously suggested in the literature [22, 38]. Collectively, these findings highlight a complex network involving microbial dysbiosis, metabolic reprogramming, and immune dysfunction, through which diabetes may lower the threshold for the onset and progression of periodontitis.

Beyond diabetes, periodontitis has been consistently linked to cardiovascular diseases (CVDs), metabolic syndrome, and atherogenic dyslipidemia. Chronic periodontal inflammation may contribute to endothelial dysfunction, recurrent bacteremia, and elevated systemic inflammatory mediators that favor atherogenesis and plaque instability. Clinical evidence linking periodontal destruction to altered lipid profiles and cardiometabolic parameters reinforces the notion that oral inflammation integrates into the pathophysiological network of metabolic syndrome [44, 45].

Furthermore, reductions in systemic inflammatory markers such as C‐reactive protein following periodontal therapy strengthen the hypothesis that periodontal control may contribute to cardiovascular risk mitigation [36]. Similarly, the risk of infective endocarditis following invasive dental procedures in high‐risk individuals underscores the systemic repercussions of oral bacterial dissemination and the need for individualized preventive strategies [46].

The association between periodontitis and CVDs has been recognized for over a decade, notably following the scientific statement from the American Heart Association in 2012, which acknowledged a consistent epidemiological relationship between these conditions [47]. Since then, this link has evolved from an observational association to a mechanistically supported paradigm, driven by advances in the understanding of microbial, inflammatory, and immunological pathways. In this context, periodontal dysbiosis, characterized by an increased abundance of Gram‐negative pathogens such as *P. gingivalis*, *T. denticola*, and *T. forsythia*, has been implicated in the initiation and propagation of systemic inflammation, oxidative stress, immune activation, and platelet aggregation, all of which are central to atherogenesis [13, 48]. Moreover, periodontal pathogens, including *Streptococcus mutans* and *P. gingivalis*, have been detected in individuals with both periodontitis and atherosclerosis, supporting a potential translocation from the oral cavity to vascular tissues [49, 50]. This process may occur through transient bacteremia and oral–gut microbial interactions, contributing to a sustained systemic inflammatory burden. In parallel, bacterial‐derived components such as LPSs and flagellins can activate key inflammatory signaling pathways, including NF‐κB and matrix metalloproteinases, thereby amplifying vascular inflammation and tissue remodeling [51]. Additional mechanisms involve virulence factors such as leukotoxin A produced by *Aggregatibacter actinomycetemcomitans*, which can disrupt neutrophil function and promote hypercitrullination, potentially linking periodontal infection to autoimmune responses and atherosclerotic processes [52]. Notably, emerging evidence also supports a bidirectional relationship, whereby cardiovascular conditions may further influence oral microbial composition through systemic inflammatory and immune‐mediated pathways [53]. Collectively, these findings reinforce the concept that periodontal dysbiosis is not only a local phenomenon but also a contributor to systemic vascular pathology.

The systemic implications of periodontitis extend to autoimmune and inflammatory conditions. Meta‐analytic evidence indicates that individuals with periodontitis have an increased risk of rheumatoid arthritis (RA; OR = 1.69; 95% CI: 1.31–2.17), likely mediated by shared immunopathogenic mechanisms, including protein citrullination induced by periodontal pathogens and subsequent autoantibody production [54]. Likewise, strong associations have been reported between periodontitis and inflammatory bowel diseases (OR = 3.17; 95% CI: 2.09–4.80), supporting the oral–gut axis hypothesis, in which oral dysbiosis may influence intestinal microbiota composition and systemic immune responses [55]. These findings suggest that periodontitis may represent a peripheral inflammatory trigger within a broader spectrum of host‐mediated chronic inflammatory disorders.

Growing evidence supports a role for oral microbial dysbiosis in the early stages of RA, particularly during its preclinical phase. Alterations in the oral microbiota have been observed in individuals at increased risk of RA, including reduced salivary microbial diversity and specific compositional shifts, such as decreased abundance of Defluviitaleaceae_UCG‐011 and *Neisseria oralis*, alongside enrichment of taxa such as *Prevotella* species [56]. Notably, subgingival dysbiosis has also been identified in anticyclic citrullinated peptide (CCP)‐positive individuals without clinical arthritis, who exhibit a higher prevalence of periodontal inflammation. In these at‐risk populations, the oral microbial profile closely resembles that observed in early RA patients, with increased abundance of genera such as *Veillonella* and *Prevotella*, suggesting that these alterations may precede and potentially contribute to disease onset [57]. Furthermore, enrichment of taxa including members of the Porphyromonadaceae family and *Saccharimonas* species has been detected prior to RA diagnosis, reinforcing the concept that specific microbial signatures may be involved in early pathogenic events [58].

Beyond local dysbiosis, the systemic dissemination of oral microorganisms has emerged as a key mechanistic link between periodontitis and RA. Periodontal pathogens—most notably *P. gingivalis*—have been detected in synovial fluid and synovial tissues of RA patients, supporting the concept that oral bacteria can translocate from the periodontal niche to distant joint sites via hematogenous routes [59]. This translocation may contribute to local immune activation and the perpetuation of synovial inflammation, particularly in genetically susceptible individuals. In addition, *P. gingivalis* and *A. actinomycetemcomitans* have been shown to induce protein citrullination and the generation of anticitrullinated protein antibodies (ACPAs), a hallmark of RA pathogenesis, thereby linking microbial dysbiosis to the breakdown of immune tolerance [59, 60]. Experimental models further support this axis, demonstrating that periodontal pathogens and their components can localize within joint tissues and induce inflammatory and arthritis‐like responses [59]. Collectively, these findings support a model in which oral dysbiosis and microbial translocation act synergistically to trigger and amplify autoimmune responses, reinforcing the biological plausibility of the association between periodontitis and RA.

Current research also highlights potential links between periodontitis and neurodegenerative diseases, including Alzheimer’s disease (AD). Observational and meta‐analytic evidence suggests an association between periodontal disease and cognitive disorders and dementia, reinforcing the role of chronic systemic inflammation as a potential shared pathway [61]. Hematogenous dissemination of periodontal pathogens and their virulence factors, systemic cytokine spillover, and microglial activation provide biological plausibility for sustained neuroinflammation and enhanced amyloid‐β (Aβ) deposition. Genetic and Mendelian randomization studies further suggest shared inflammatory and molecular pathways linking periodontitis, CVDs, and neurodegeneration [62]. Collectively, these interconnections reinforce the concept that periodontitis functions as a systemic inflammatory burden capable of modulating distant organ systems.

Emerging evidence suggests that periodontitis may also play a contributory role in neurodegenerative disorders, particularly AD. Initially conceptualized as a disorder driven predominantly by amyloid deposition and neuronal loss, AD is now increasingly understood within a broader framework that incorporates chronic inflammation and microbial influences. In this context, a growing body of literature supports a bidirectional relationship between oral microbial dysbiosis and AD pathology, suggesting the existence of a self‐reinforcing cycle involving systemic inflammation, neuroinflammation, and microbial imbalance [63–65]. Periodontal pathogens—most notably *P. gingivalis*—and their virulence factors, such as gingipains, have been identified in the brains of individuals with AD, supporting the hypothesis of microbial translocation from the oral cavity to the central nervous system [64]. Interestingly, Aβ has also been proposed to function as an antimicrobial peptide, indicating that its accumulation may represent, at least in part, a host defense response to chronic microbial challenges [66, 67].

Recent advances have further refined this model by identifying key oral pathogens, including *P. gingivalis*, *T. denticola*, and *F. nucleatum*, as potential drivers of AD through mechanisms involving gingipain‐induced Aβ aggregation, systemic inflammation, and disruption of the blood–brain barrier [68].Notably, emerging data suggest that specific oral microorganisms, such as *Veillonella* in saliva and *P. gingivalis* in gingival crevicular fluid, may serve as noninvasive biomarkers associated with increased AD risk, reinforcing the clinical relevance of the oral–brain axis. These findings are complemented by experimental evidence demonstrating that oral pathobiont exposure can induce neuroinflammation, neuronal damage, and amyloid deposition [69]. In addition, the identification of biomarkers capable of early detection of the risk for developing AD and their correlation with periodontitis represents a promising strategy for the integrated management of both conditions. The use of microbiological, inflammatory, and metabolic markers may not only contribute to early diagnosis and risk stratification but also assist in monitoring therapeutic responses. In this context, the association between periodontal health and neurological outcomes reinforces the importance of preventive and long‐term maintenance approaches, promoting greater patient adherence to treatment and enhancing the understanding of the systemic impact of periodontitis.

### 2.5. Treatment Perspectives

The current understanding of the etiopathogenesis of periodontal diseases enables the development of alternative management strategies. While basic periodontal treatment, which involves mechanical debridement, remains broadly effective for most cases [70], approaches such as host response modulation and modifications in the structural microarrangements of the oral microbiome seem to be valid alternatives for treating refractory periodontitis [71].

Regarding the different microbial profiles identified through genetic sequencing methods, it has been suggested that these data could serve as a standard for future diagnostic use, given their role in identifying other inflammatory diseases in longitudinal studies described in the literature [72–74]. However, there is still a need for studies based on larger populations to enable reliable pattern recognition.

From the perspective of innovative treatments, using prebiotics and probiotics has shown promising results in in vitro studies; however, more confirmation through clinical trials and cohort studies is necessary to support their use [75]. Host response modulation with resolvins also seems to be a promising therapeutic approach based on recent human studies [71].

Systemic antimicrobials now have clearer evidence‐based indications, showing greater benefits in patients over 55 years of age with generalized periodontitis and probing depths exceeding 5 mm [76]. Nevertheless, prescription should not rely solely on age but rather on clinical parameters such as the degree of inflammation, disease stability, bleeding sites, and risk of progression. Indiscriminate use poses significant risks, including antimicrobial resistance and systemic adverse effects such as gastrointestinal disturbances, allergic reactions, and drug interactions [77, 78]. For these reasons, antimicrobial therapy in periodontics must be carefully individualized, ensuring that potential benefits outweigh the risks [79, 80].

In the context of advances in periodontitis management, a recent study proposed a new criterion for defining disease remission or control. In this regard, Feres et al. [30] suggest that the presence of up to four sites with probing depths ≥5 mm, provided bleeding on probing (BOP) occurs in less than 10% of sites, may be considered an adequate clinical indicator—the so‐called “End Point.” This proposal aligns with the definition of stable, remitted periodontitis as proposed by Dietrich et al. [81] in 2018. Additionally, beyond the staging and grading of periodontitis proposed by the classification system [1, 82, 83], Dietrich et al. [81] recommended establishing the current periodontal health/disease status based on probing depth and the percentage of sites with bleeding. According to this consensus, periodontal status posttreatment is classified based on objective clinical parameters: periodontitis is considered stable when there is <10% BOP and all probing depths are ≤4 mm, without bleeding at 4 mm sites. Periodontal remission refers to the absence of active inflammation, even in a reduced periodontium, with controlled probing depths of ≤4 mm. This includes no BOP at sites of 4 mm but with a BOP score of more than 10% overall. The disease is considered unstable when there are bleeding sites ≥4 mm or any PPD ≥5 mm, indicating active inflammation and a risk of progression. This approach emphasizes inflammation control as the primary clinical marker of stability. Following diagnosis, it is equally important to determine the patient’s risk factor profile [81].

This set of criteria marks a significant clinical advancement by establishing a feasible therapeutic goal aligned with the current understanding of periodontal disease causes. It is important to note that the microbiota associated with periodontitis remains different from that in healthy individuals, even after controlling the disease. Therefore, defining periodontal health as a complete return to an intact periodontium may not be realistic. Additionally, using BOP as a clinical parameter is promising, supported by studies emphasizing the importance of inflammation in periodontal disease progression [80, 84–86].

Overall, the convergence of microbiological dysbiosis, immune activation, and metabolic dysregulation situates periodontitis as a relevant component of the pathobiology of chronic systemic comorbidities. This integrative model underscores the need for interdisciplinary strategies to control inflammation beyond the oral environment and supports the inclusion of periodontal care within comprehensive approaches to chronic disease prevention and management.

Although adjunctive approaches such as photodynamic and laser therapies show short‐term metabolic and periodontal benefits, their clinical relevance requires confirmation through well‐designed long‐term trials [87, 88].

## 3. Conclusion

The understanding of periodontal disease has significantly progressed over the past decades, shifting from a focus on specific microorganisms to a broader model that considers the ecological complexity of the oral microbiome and its interactions with the host. Recent evidence shows that periodontitis results from a synergistic and dysbiotic microbial succession, driven by changes in the oral microenvironment and influenced by systemic and behavioral factors.

Advances in genomics and metabolomics have enabled the identification of distinct microbiome profiles associated with periodontal health and disease, emphasizing the roles of core bacteria and keystone pathogens. These findings not only reaffirm the multifactorial nature of the disease but also pave the way for innovative therapeutic approaches, such as host response modulation and the use of probiotics and prebiotics.

From a clinical perspective, although mechanical debridement remains the standard treatment, integrating new strategies may offer a significant step forward, especially in refractory cases. Redefining the criteria for therapeutic success to emphasize clinical stability and inflammation control (BOP), as well as considering patient‐specific risk factors, better aligns with our current understanding of the etiopathogenesis of periodontal disease.

Importantly, periodontitis should also be understood within the context of its systemic implications. A growing evidence links periodontal inflammation and dysbiosis to a range of systemic conditions, including T2DM, CVDs, autoimmune disorders, and neurodegenerative diseases. These associations are supported by shared inflammatory pathways, immune dysregulation, and, in some cases, microbial translocation, highlighting a bidirectional relationship in which systemic conditions may also exacerbate periodontal breakdown. This perspective reinforces the concept of periodontitis as part of a broader network of chronic inflammatory diseases, with potential implications for interdisciplinary prevention and management strategies.

## Funding

No funding was received for this manuscript.

## Disclosure

All AI‐assisted outputs were critically reviewed and validated by the authors, who take full responsibility for the accuracy and scientific interpretation of the content.

## Conflicts of Interest

The authors declare no conflicts of interest.

## Data Availability

Data sharing is not applicable to this article, as no datasets were generated or analyzed during the current study.
